# LRRC8A is essential for volume‐regulated anion channel in smooth muscle cells contributing to cerebrovascular remodeling during hypertension

**DOI:** 10.1111/cpr.13146

**Published:** 2021-11-01

**Authors:** Xiang‐Yu Li, Xiao‐Fei Lv, Cheng‐Cui Huang, Lu Sun, Ming‐Ming Ma, Canzhao Liu, Yong‐Yuan Guan

**Affiliations:** ^1^ Department of Pharmacology Zhongshan School of Medicine Sun Yat‐sen University Guangzhou China; ^2^ Key Laboratory of Drug Monitoring and Control Drug Intelligence and Forensic Center Ministry of Public Security Beijing China; ^3^ Department of Pharmacy the Sixth Affiliated Hospital of Sun Yat‐Sen University Guangzhou China; ^4^ Department of Pharmacy Division of Life Sciences and Medicine the First Affiliated Hospital of USTC University of Science and Technology of China Hefei China; ^5^ Department of Cardiovascular Medicine Translational Medicine Research Center Zhujiang Hospital Southern Medical University Guangzhou China

## Abstract

**Objectives:**

Recent studies revealed LRRC8A to be an essential component of volume‐regulated anion channel (VRAC), which regulates cellular volume homeostasis. However, evidence for the contribution of LRRC8A‐dependent VRAC activity in vascular smooth muscle cells (VSMCs) is still lacking, and the relevant functional role of LRRC8A in VSMCs remains unknown. The primary goal of this study was to elucidate the role of LRRC8A in VRAC activity in VSMCs and the functional role of LRRC8A in cerebrovascular remodeling during hypertension.

**Materials and Methods:**

siRNA‐mediated knockdown and adenovirus‐mediated overexpression of LRRC8A were used to elucidate the electrophysiological properties of LRRC8A in basilar smooth muscle cells (BASMCs). A smooth muscle–specific overexpressing transgenic mouse model was used to investigate the functional role of LRRC8A in cerebrovascular remodeling.

**Results:**

LRRC8A is essential for volume‐regulated chloride current (*I*_Cl, Vol_) in BASMCs. Overexpression of LRRC8A induced a voltage‐dependent Cl^−^ current independently of hypotonic stimulation. LRRC8A regulated BASMCs proliferation through activation of WNK1/PI3K‐p85/AKT axis. Smooth muscle‐specific upregulation of LRRC8A aggravated Angiotensin II‐induced cerebrovascular remodeling in mice.

**Conclusions:**

LRRC8A is an essential component of VRAC and is required for cell volume homeostasis during osmotic challenge in BASMCs. Smooth muscle specific overexpression of LRRC8A increases BASMCs proliferation and substantially aggravates basilar artery remodeling, revealing a potential therapeutic target for vascular remodeling in hypertension.

## Graphical abstract

The schematic diagram for LRRC8A role in cerebrovascular remodeling. LRRC8A is an essential component of VRAC in BASMCs. During the challenge of hypertension, the activated LRRC8A channel‐mediated‐Cl^−^ efflux increases WNK1 phosphorylation, which in turn triggers AKT phosphorylation and promotes BASMCs proliferation, eventually exacerbates hypertension‐induced cerebrovascular vascular remodeling.

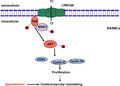

## 1 INTRODUCTION

Cell volume homeostasis is essential for many biological processes involved in vertebrate heath and survival.^1^, ^2^ The decreased extracellular or increased intracellular osmolarity under hypotonic stress will evoke the process of regulatory volume decrease (RVD), which involves the activation of the channel‐mediated Cl^−^ and K^+^ transport as well as taurine efflux.^3^, ^4^ Volume‐regulated anion channel (VRAC) is ubiquitously expressed in eukaryotic cells and is involved in many physiological and pathophysiological functions.^5^, ^6^, ^7^, ^8^, ^9^ Although the physiological and pharmacological properties of VRAC have been described in detail over the past 30 years, the precise molecular identity is still controversial. Previous studies have identified some candidates such as P‐glycoprotein, ClC‐3, and Bestrophin‐1 as key components required for VRAC activity and the RVD process.^10^, ^11^, ^12^ However, the sufficiency of these candidates for VRAC activity was not fully verified in all cell types by the subsequent studies. LRRC8A, a leucine‐rich repeat‐containing protein, was recently identified as an essential component of VRAC using an unbiased Genome‐wide RNAi Screen in many cell types including HEK293T and T‐lymphocytes.^13^, ^14^ However, Andrea Milenkovic et al. found that Bestrophin 1, but not LRCC8A, was indispensable for cell volume regulation in retinal pigment epithelium (RPE) cells differentiated from human‐induced pluripotent stem cells.^15^ Another study also demonstrated that LRRC8A was not essential for VRAC activation in HeLa cells,^16^ suggesting that the contribution of LRRC8A to cell volume regulation and the molecular identity of VRAC are variable in different cell types or tissues.

Vascular remodeling is the alteration of vascular structure, and it is characterized by the thinning of the lumen diameter inside and outside the blood vessels and increased tube wall area during the development of hypertension. Vascular remodeling is an active process in response to the development of hypertension and subsequently contributes to the pathophysiology of cardiovascular diseases such as stroke and myocardial infarction.^17^, ^18^, ^19^ Our previous study found that the activity of VRAC enhanced cell proliferation in hypertensive rat basilar smooth muscle cells (BASMCs) and cerebrovascular remodeling, suggesting that VRAC is involved in vascular remodeling process during chronic hypertension.^20^, ^21^ Moreover, we also demonstrated that Simvastatin could attenuate rat cerebrovascular remodeling via inhibition of VRAC,^22^ suggesting that VRAC is a promising therapeutic target for vascular remodeling. Particularly, we previously demonstrated ClC‐3 as a potential molecular identity of VRAC, which is at least partially contributing to cell volume regulation in smooth muscle cells and involves in the process of cerebrovascular remodeling during hypertension.^23^, ^24^ Given the complexity and controversy of VRAC subunit composition in different tissues and based on our previous studies, we hypothesize that LRRC8A is an essential component of VRAC in smooth muscle cells, and that LRRC8A‐mediated BASMCs proliferation is required for cerebrovascular remodeling during hypertension. Here, we found that LRRC8A is indispensable for volume regulation in BASMCs. We also provided evidence that LRRC8A regulates the process of BASMCs proliferation through phosphorylation of lysine‐deficient protein kinase 1 (WNK1), and that the smooth muscle‐specific overexpression of LRRC8A aggravates Angiotensin II‐induced cerebrovascular remodeling in mice.

## 2 MATERIALS AND METHODS

The sources of reagents are given in Table S1. All experimental procedures were approved by the Sun Yat‐sen University Animal Care and Use Committee (SYSU‐IACUC‐2020‐000446) and were in accordance with the “Guide for the Care and Use of Laboratory Animals” issued by the Ministry of Science and Technology of China and the current NIH guidelines.

### 2.1 Cell culture

Four‐week‐old healthy male Sprague‐Dawley rats (80–100 g) were used for BASMCs isolation. Rat BASMCs were isolated and cultured from rat basilar arteries using the method as described previously.^22^ BASMCs were incubated in Dulbecco's Modified Essential Medium (DMEM)/F‐12 supplemented with 20% fetal bovine serum (FBS). Passages 8–12 of BASMCs were used for experiments. A10 vascular smooth muscle cells (American Type Culture Collection) were cultured in DMEM/F‐12 containing 10% Newborn Calf Serum (NCS) as described before.^25^

### 2.2 siRNA transfection

Gene silencing was performed using gene‐specific small interfering RNA (siRNA) as described before.^26^ The sequences of siRNA against rat LRRC8A and WNK1 were synthesized by InvitrogenTM (Life Technologies). The sequences were Rat LRRC8A, 5′GCCUGCAUUGGUUUGCCAATT 3′ and Rat WNK1, 5′GGUGUCGGCAAAUCCUUAATT3′. BASMCs were transfected with negative siRNA (scrambled siRNA), LRRC8A siRNA, or WNK1 siRNA in serum‐free‐medium using Hiperfect Transfection Reagent (Qiagen). Following 4–6 h of incubation, fresh medium containing 20% FBS was added to BASMCs for another 48 h.

### 2.3 Adenovirus‐mediated overexpression

LRRC8A full length and LRRC8A LRRD‐Del containing adenovirus (Ad‐LRRC8A and Ad‐LRRC8A Del) were produced by Sunbio Medical Biotechnology. The LRRC8A‐GFP vector was a gift from Dr. Tobias Stauber (MDC/FMC Institute, Berlin, Germany). The LRRC8A‐FLAG plasmid was a gift from Dr. Zhaozhu Qiu (Howard Hughes Medical Institute, USA). Adenovirus transduction was performed according to the protocol as previously described.^27^ BASMCs were transduced with adenovirus when the cells reached 50% confluence.

### 2.4 Western blot

Western blot was performed as described before.^28^ Briefly, tissues or cells were lysed with RIPA lysis buffer containing protease inhibitor cocktail (Merk). Proteins were separated by 8–12% SDS‐PAGE and transferred to PVDF membranes. After blocking with 5% milk at room temperature for 1 h, blots were incubated overnight with primary antibodies at 4°C and then incubated for 1 h with secondary antibodies at room temperature. Bands were detected with Pierce ECL western blotting substrate and quantified with the computer‐aided 1‐D gel imaging system (Bio‐Rad).

### 2.5 Electrophysiological experiments

VRAC current was recorded with an Axopatch 200B Amplifier (Axon Instrument) using a whole‐cell patch clamp technique as described previously.^23^, ^29^ The current‐voltage curve was held at −40 mV and applied from −100 to −120 mV for 200 ms in −20 mV increments at an interval of 5 s. The data were stored on a computer after digitalization at 5 kHz by Digidata1500A (Axon Instrument). The hypotonic solution contained 107 mM *N*‐methyl‐d‐glucamine chloride (NMDG‐Cl), 1.5 mM MgCl_2_, 2.5 mM MnCl_2_, 0.5 mM CdCl_2_, 10 mM glucose, and 10 mM HEPES, with pH adjusted to 7.4 by NMDG. The osmotic pressure was adjusted to 230 mOsm/kg, and the osmotic pressure was measured by a freezing point depression osmometer (OSMOMAT030). The isotonic solution was prepared by adding 70 mM d‐mannitol to the hypotonic solution and adjusted to 300 mOsm/kg osmotic pressure. A 370 mOsm/kg hypertonic bath solution was prepared by adding 140 mM d‐mannitol to the hypotonic solution. All experiments were performed at room temperature.

### 2.6 Measurement of [Cl^−^]_i_

The recording of [Cl^−^]_i_ fluorescence quenching was measured by 6‐methoxy‐*N*‐ethylquinolinium iodide (MEQ) as described previously.^20^ Briefly, cells were incubated with 100–150 μM 6‐methoxy‐*N*‐ethyl‐1,2‐dihydroquinoline (dihydro‐MEQ) in Ringer's buffer solution containing 119 mM NaCl, 2.5 mM KCl, 1.0 mM NaH_2_PO4, 1.3 mM MgSO_4_, 2.5 mM CaCl_2_, 26 mM NaHCO_3_, and 11 mM glucose, pH 7.4, at room temperature in the dark for 30 min. Fluorescence quenching induced by Cl^−^ was monitored by MetaFluor Imaging software (Universal Imaging Systems) with 350‐nm excitation wavelength and 435‐nm emission wavelength. Relationship between fluorescence intensity of MEQ and chloride concentration was given by the Stern–Volmer equation: (*F*_O_/*F*) – 1 = *K*_SV_ [*Q*], where *F*_O_ is the fluorescence intensity without halide or other quenching ions; *F* is the fluorescence intensity in the presence of quencher; [*Q*] is the concentration of quencher; and *K*_SV_ is the Stern–Volmer constant.

### 2.7 Cell viability assay

Cell viability was measured by a Cell Counting Assay Kit‐8 (CCK‐8; Dojindo Molecular Technologies) according to our established method.^30^ Briefly, 2 × 10^3^ BASMCs were seeded in a 96‐well plate and preincubated in a humidity chamber for 24 h. Ten microliters of CCK‐8 solution was added to each well and incubated for 2 h, after which the absorbance at 450 nm was measured using a microplate reader.

### 2.8 BrdU incorporation assay

Cell proliferation was assessed by 5‐bromo‐2′‐deoxyuridine (BrdU) incorporation as described previously.^31^, ^32^ Cells were trypsinized and plated into 96‐well culture plates at a density of 1 × 10^5^ to 1.5 × 10^5^ cells per well in DMEM/F‐12 medium supplemented with 10% NCS. Cell number was determined in triplicate using a hemocytometer. After 24 h, cells were rendered quiescent in 0.5% NCS for 24 h. 10 μmol/L BrdU was added to the medium for 18 h and then anti‐BrdU peroxidase was added for 1 h. The conjugate was removed by washing three times with 200–300 μl wash buffer per well. A total of 100 μl of substrate solution was added and incubated for 10 min, after which 25 μl 1 mol/L H_2_SO_4_ was added. The incorporation was measured at 450 nm on an ELISA microplate reader (BIO‐TEK synergy HT, US).

### 2.9 Flow cytometry

Flow cytometry was used to detect the population fraction of BASMCs which were collected by Propidium Iodide (PI) Cell Cycle Detection Kit (KeyGEN Biotech) as described previously.^33^ BASMCs were treated according to the manufacturer's instructions when collected by centrifugation at 2000 *g* for 5 min. Then the samples were analyzed by flow cytometry.

### 2.10 LRRC8A smooth muscle‐specific transgenic mice

The tissue‐specific LRRC8A transgenic LoxP line was produced by Cyagen as described before.^34^ Briefly, the fertilized eggs of C57BL/6 mice were transfected with the targeting carrier containing the CAG‐LoxP‐Stop‐LoxP‐LRRC8A construct. Then, LRRC8A LoxP line was crossed with the Tagln‐Cre line (STOCK Tg (Tagln‐cre) 1Her/J, the Jackson Laboratory) to generate LRRC8A smooth muscle specific‐overexpressed mice (LRRC8A^SOE^). Mice with the Tagln‐Cre promoter without LoxP site were considered as the control mice (Ctrl). LRRC8A^SOE^ and Ctrl were genotyped by PCR on tail DNA using primers as shown in Table S2.

### 2.11 Animal models of hypertension

The Ang II‐induced hypertensive Animal model was implemented with an osmotic pump (Alzet model, 1004) filled with Ang II in saline solution as described previously.^34^ Mice in control group were implanted with an osmotic pump with normal saline only. The 2k2c hypertensive rats were prepared as described previously.^35^

### 2.12 Immunostaining

Mice were perfusion‐fixed at a constant pressure (100 mmHg) via the left ventricle with Krebs solution, followed by 4% freshly depolymerized paraformaldehyde for 10 min. The brains were carefully removed, and sections (8 µm) of freshly frozen basilar arteries were prepared as described before.^26^ Immunofluorescence staining was carried out using specific primary and secondary antibodies as described before.^36^ All the images were scanned by a Zeiss microscope (Axio Imager Z1).

### 2.13 Electron microscopy

The mice were perfusion‐fixed as described before.^24^ Brains were removed, and tissue blocks containing the basilar artery at midpoint were cut into cubes of 1 × 1 × 3 mm. Ultrathin sections at 80–100 nm were prepared and stained with uranyl acetate and lead citrate, which were then viewed under a transmission electron microscope (FEI TECNAI spirit G2).

### 2.14 Statistical analysis

All statistical analyses were performed using GraphPad Prism 5 (GraphPad Software). Data were expressed as means ± SE. Statistical significance was determined by Student's *t* test or ANOVA followed by the Bonferroni multiple comparison test. *N* represented the number of independent experiments performed with different batches of cells or different mice. A value of *p* < 0.05 was considered statistically significant.

## 3 RESULTS

### 3.1 LRRC8A is essential for VRAC channel activity in BASMCs

**FIGURE 1:**
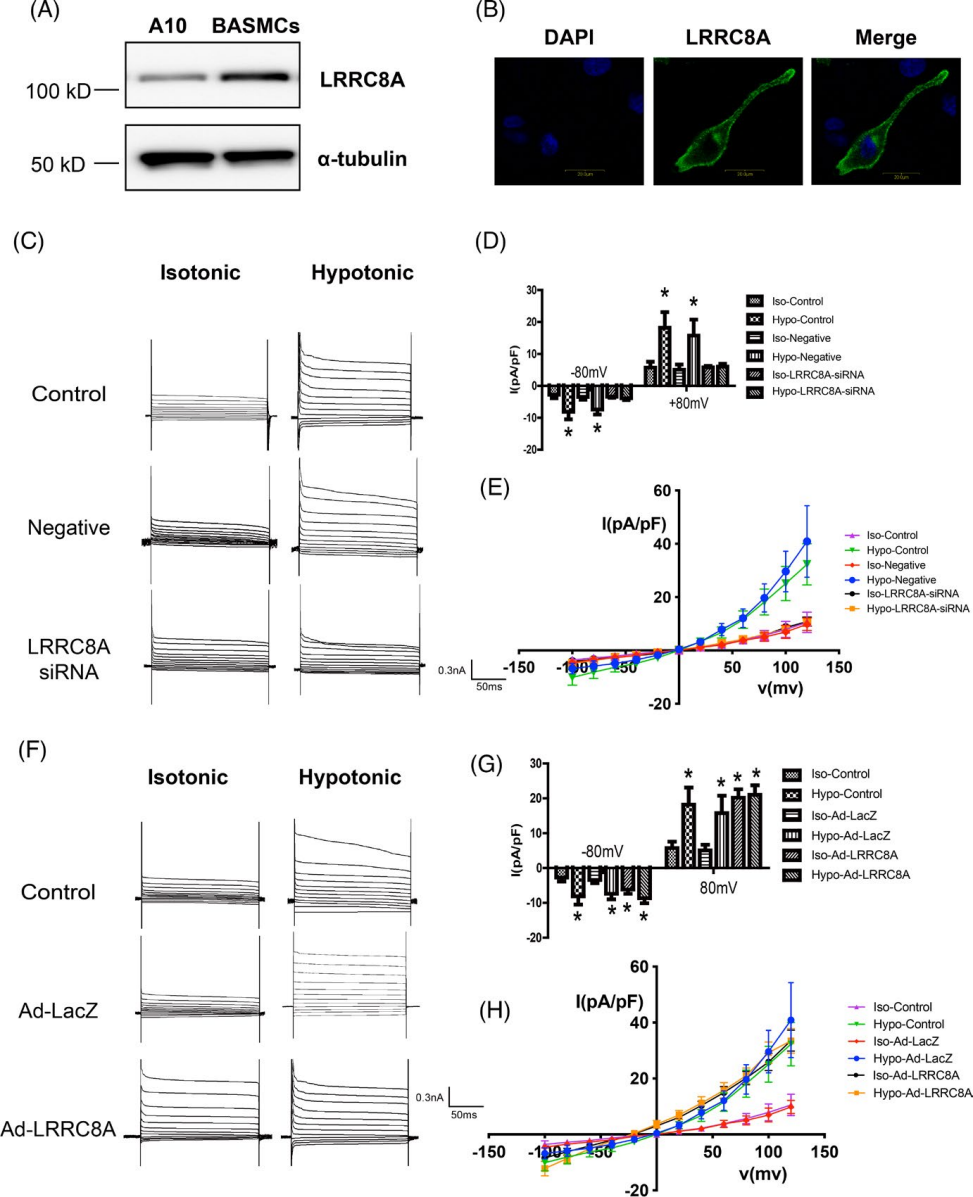
LRRC8A is necessary for VRAC. (A) Representative western blot images showing the expression of LRRC8A in A10 cells and BASMCs. (B) Representative confocal images showing the localization of LRRC8A in BASMCs. Scale, 200 μm. (C–E) Representative traces of VRAC current (C), average current densities measured at ±80 mV (D), and *I*–*V* curves (E) in Control, siRNA negative control (Negative), or LRRC8A siRNA‐transfected BASMCs (*n* = 6, *p < 0.05 VS Control or Negative). (F–H) Representative traces of VRAC current (F), average current densities measured at ±80 mV (G), and *I*–*V* curves (H) in Control, Lacz adenovirus (Ad‐Lacz), or LRRC8A‐expressing adenovirus (Ad‐LRRC8A)‐transfected BASMCs (*n* = 6, *p < 0.05 VS Control or Ad‐LacZ)

To investigate the association between LRRC8A and VRAC activity in VSMCs, we first examined the endogenous expression and localization of LRRC8A in A10 vascular smooth muscle cell line and freshly isolated BASMCs. Western blot results showed that LRRC8A was widely expressed in both A10 cells and BASMCs (Figure 1A). Immunostaining using FITC‐labeled LRRC8A antibody showed that LRRC8A was predominantly expressed on cell surface in BASMCs (Figure 1B).

To determine whether LRRC8A is essential for VRAC activity in BASMCs, we directly recorded hypotonic solution‐induced VRAC currents by whole‐cell patch‐clamp recording in BASMCs. We effectively silenced the endogenous expression of LRRC8A in BASMCs by a specific siRNA (Figure S1). The results showed that knockdown of LRRC8A significantly decreased hypotonic solution‐induced Cl^−^ current in BASMCs (Figure 1C). The current density reduced from −6.80 ± 0.90 pA/pF to −3.84 ± 0.61 pA/pF at −80 mV and 19.04 ± 3.40 pA/pF to 6.63 ± 0.87 pA/pF at +80 mV, respectively (Figure 1D,E). It has been reported that overexpression of LRRC8A in HEK293T and HeLa cells did not enhance the *I*_Cl, Vol_ current density.^13^ Consistent with that previous study, we also found that adenovirus‐mediated overexpression of LRRC8A in BASMCs could not further increase the density of hypotonic solution–induced current (Figure 1F–H, Figure S2). However, we unexpectedly found that the overexpression of LRRC8A induced an enlarged outwardly rectifying current in isotonic solution; the current density was increased from −3.09 ± 0.67 pA/pF to −6.18 ± 1.20 pA/pF at −80 mV and 5.39 ± 1.10 pA/pF to 20.19 ± 2.39 pA/pF at 80 mV, respectively, in the isotonic bath solutions.

### 3.2 Overexpression of LRRC8A induced a voltage‐dependent Cl^−^ current independently of hypotonicity stimulation

**FIGURE 2:**
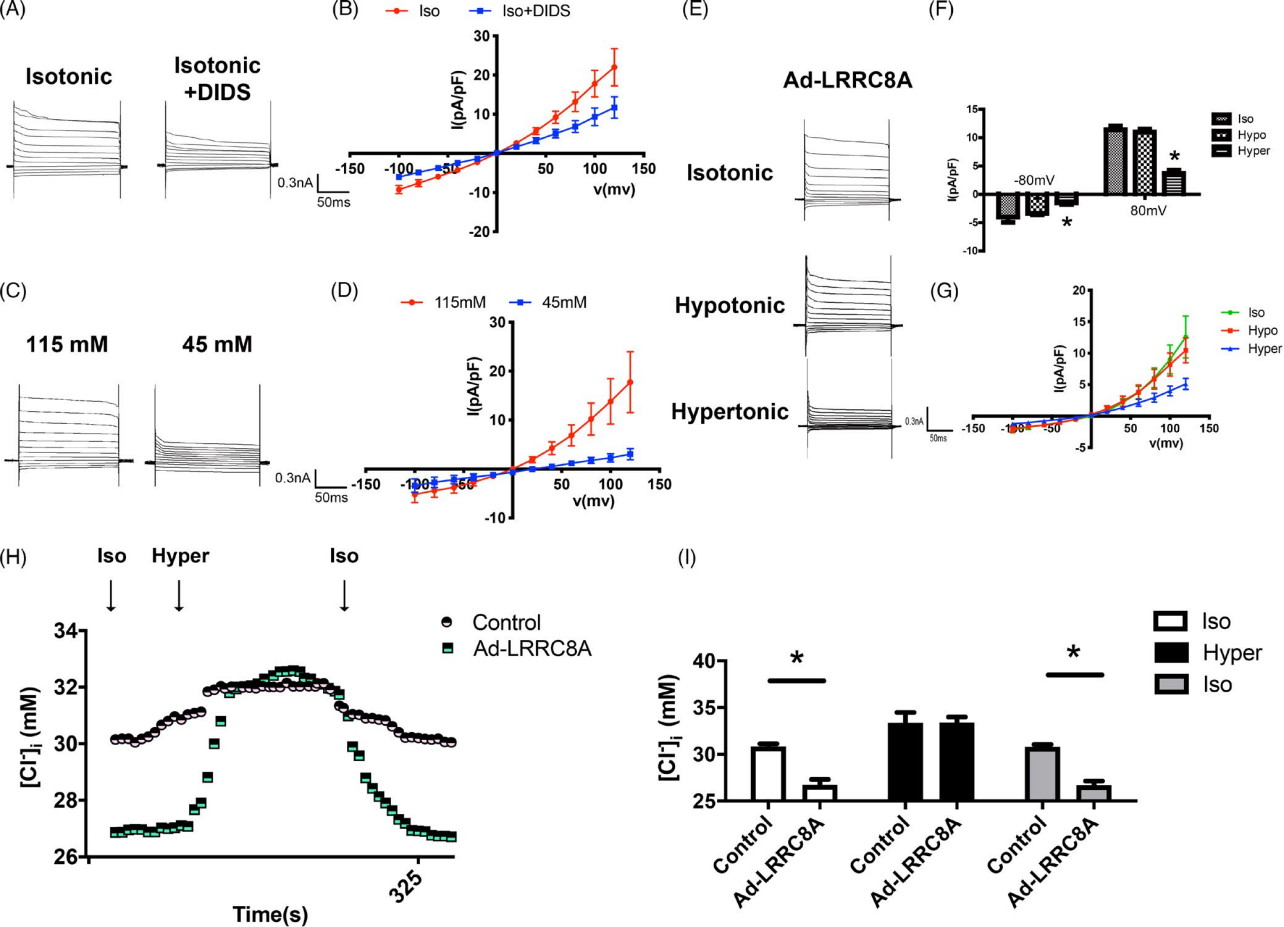
Overexpression of LRRC8A induced a voltage‐dependent Cl^−^ current under isotonic conditions. (A, B) Representative traces of current (A) and *I*–*V* curves (B) in LRRC8A‐expressing adenovirus‐transfected BASMCs before and after DIDS treatment in isotonic solution (*n* = 6). (C, D) Representative traces of current (C) and *I*–*V* curves (D) when extracellular Cl^−^ concentration was changed from 115 to 45 mM in LRRC8A‐expressing adenovirus‐transfected BASMCs (*n* = 6). (E–G) Representative traces of current (E), average current densities measured at ±80 mV (F), and *I*–*V* curves (G) in LRRC8A overexpression adenovirus‐transfected BASMCs showing the evoked voltage‐dependent Cl^−^ current at isotonic condition were inhibited after exposure to hypertonic solution (*n* = 5, *p < 0.05 VS ISO). (H) Cl^−^ concentration was measured in the indicated solutions in Control or LRRC8A‐expressing adenovirus (Ad‐LRRC8A)‐transfected BASMCs. (I) Quantification of Cl^−^ concentration from (H) in the indicated solutions (*n* = 6, *p < 0.05)

To better understand the electrophysiological properties of LRRC8A mediated current, DIDS (4,4′‐diisothiocyano‐2,2′‐stilbenedisulfonic acid, disodium salt), a chloride channel blocker was used to detect the property of the current. DIDS dramatically inhibited the current induced by the overexpression of LRRC8A in isotonic solution (Figure 2A,B). In addition, this outwardly rectifying current reverse potential was shifted from 2e‐3 ± 0.56 mV to 21.80 ± 0.77mV, whereas extracellular Cl^−^ concentration was changed from 115 to 45 mM, as calculated by the Nernst equation (Figure 2C,D). Meanwhile, the reverse potential induced by Br^−^ was −8.33 ± 2.32 mV, which was more negative compared with Cl^−^ induced reverse potential (−2.40 ± 1.30 mV). Aspartate activated a positive reverse potential of 17.81 ± 4.55 mV, indicating the permeability preference of Br^−^ > Cl^−^ > Asp^−^, similar to that of VRAC (Table S3). To further examine the response of the current to osmotic pressure, we changed the isotonic solution to hypertonic solution. Interestingly, the hypertonic bath solution blocked the current induced by overexpressing LRRC8A under isotonic solution (Figure 2E–G). These results suggested that overexpressing LRRC8A under isotonic solution evoked an Cl^−^ current with a similar characteristic of VRAC. We directly measured Cl^−^ concentration ([Clˉ]_i_) and found that overexpression of LRRC8A significantly reduced [Cl^−^]_i_ in isotonic solution. Alteration of the medium from isotonic solution to hypertonic solution reversed the decrease in [Cl^−^]_i_ induced by overexpression of LRRC8A (Figure 2H,I).

### 3.3 Leucine‐rich repeats domain is necessary for LRRC8A‐activated Cl^−^ current in isotonic solution

**FIGURE 3:**
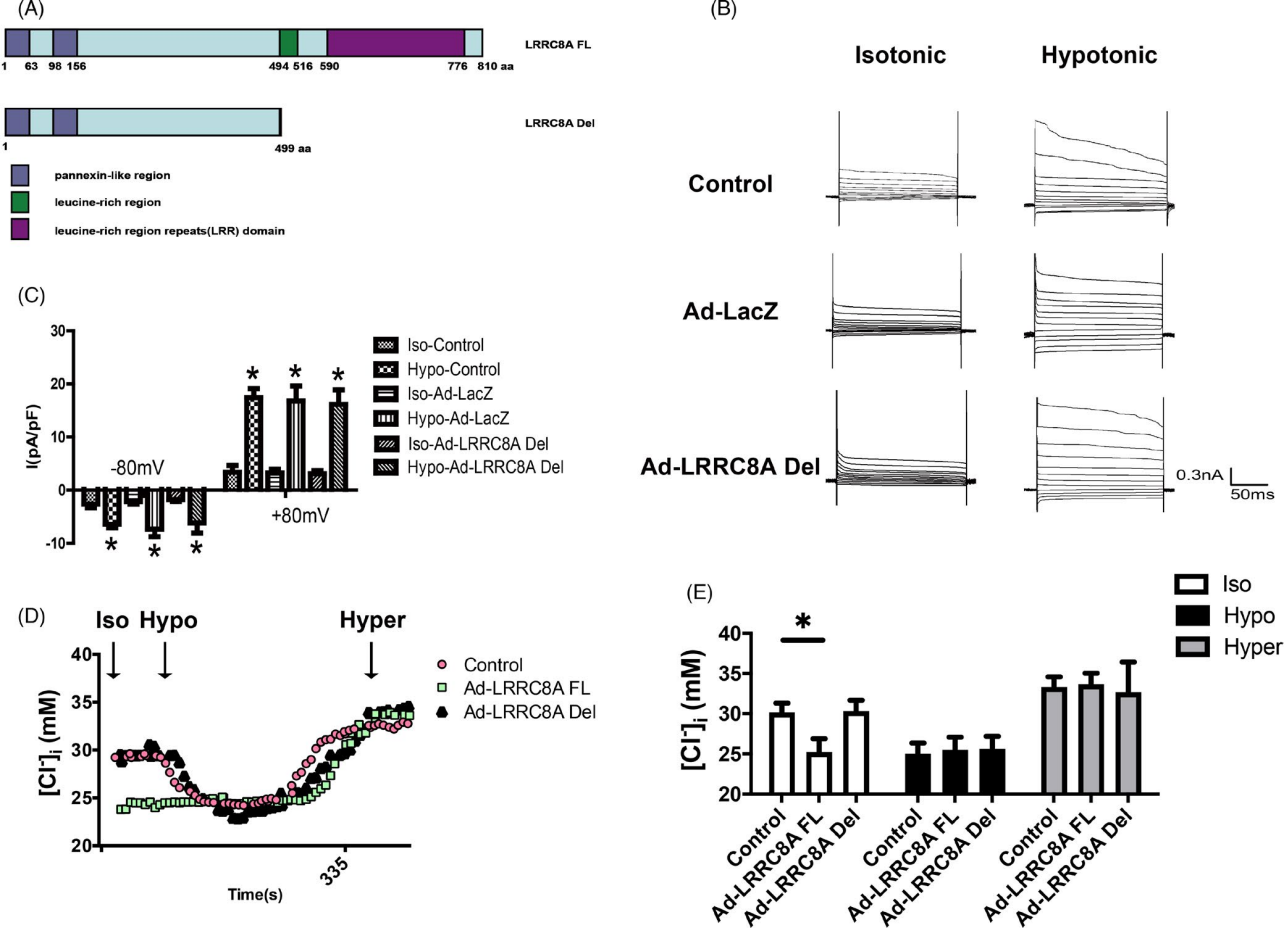
LRRD is necessary for LRRC8A‐activated Cl^−^ current in isotonic solution. (A) Schematic drawing of the structures of LRRC8A full length (LRRC8A‐FL) and LRRD deletion (LRRC8A Del). (B, C) Representative traces of current (B) and average current densities measured at ±80 mV (C) in Control, Lacz adenovirus (Ad‐Lacz), or LRRD deletion (LRRC8A Del) adenovirus‐transfected BASMCs (*n* = 6, *p < 0.05 VS Control or Ad‐LacZ). (D) Cl^−^ concentration was measured in the indicated solutions in Control, LRRC8A‐expressing adenovirus (Ad‐LRRC8A FL), or LRRD deletion (LRRC8A Del) adenovirus‐transfected BASMCs in the indicated solutions. (E) Quantification of Cl^−^ concentration from (D) in the indicated solutions (*n* = 6, *p < 0.05)

LRRC8A is a protein consisting of four transmembrane helices in its N‐terminal region, followed by leucine‐rich repeats domain (LRRD) in the middle and its C‐terminal regions. It has been demonstrated that N‐terminal transmembrane pore domain is essential for channel activity.^13^, ^37^ However, the function of LRRD remains unclear. We constructed a truncated LRRC8A, in which the C‐terminal LRRD was deleted, and transiently expressed this construct in BASMCs (Figure 3A). Whole‐cell patch‐clamp recording showed that transient expression of LRRD‐truncated LRRC8A did not induce larger VRAC current compared to the control group during hypotonic stimulation. Interestingly, unlike LRRC8A full length protein, the Cl^−^ current triggered by LRRC8A overexpression in the absence of hypotonic stimulation was not observed in LRRD‐truncated mutant transfected cells (Figure 3B,C). Consistent with the current recording data, chloride concentration was not altered in LRRD‐truncated mutant transfected cells (Figure 3D,E). These data suggest that LRRD was indispensable for this voltage‐dependent Cl^−^ current in the absence of hypotonicity stimulation.

### 3.4 LRRC8A promotes proliferation in BASMCs

**FIGURE 4:**
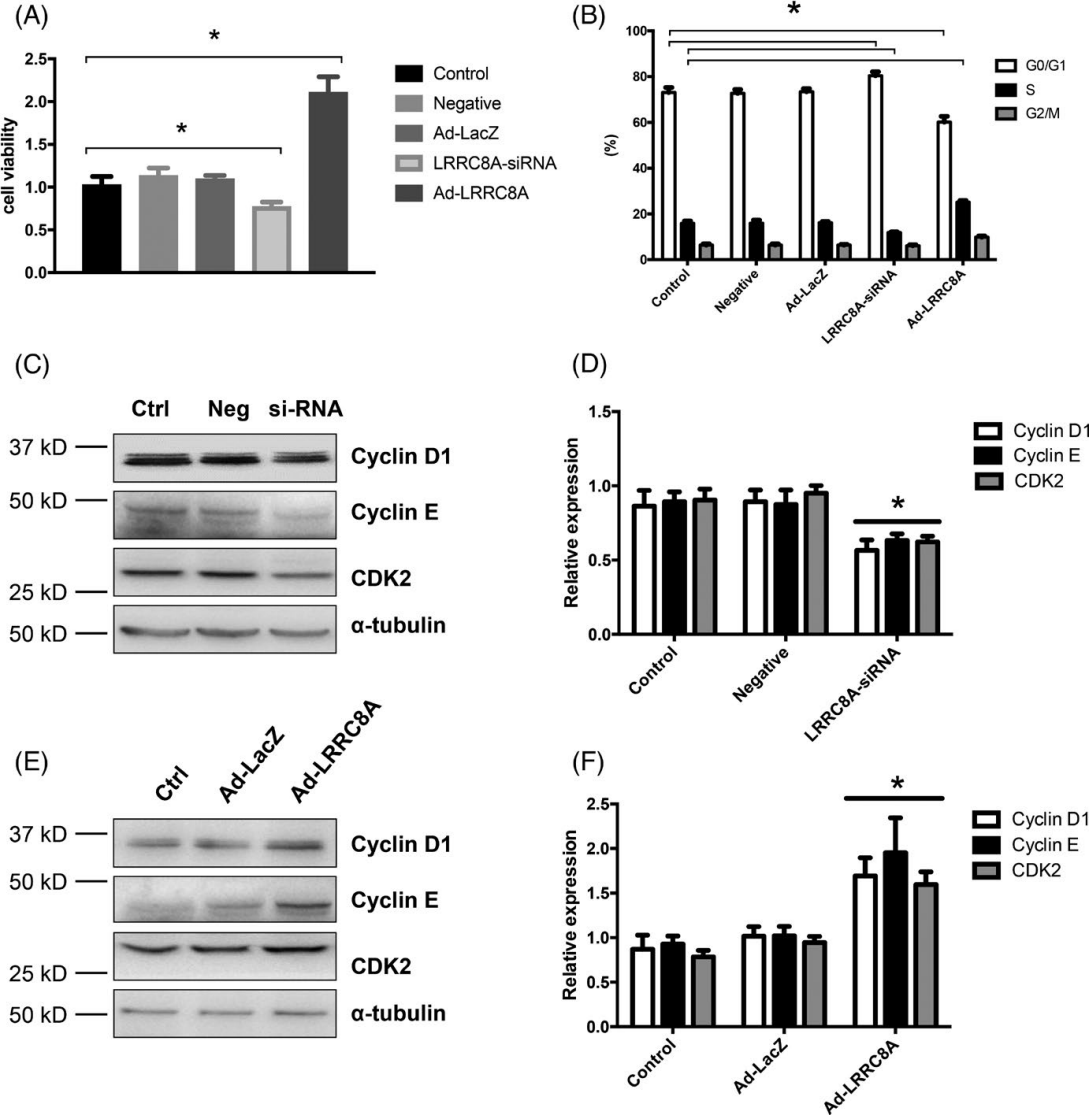
LRRC8A increases proliferation in BASMCs. BASMCs were transfected with LRRC8A siRNA, LRRC8A‐expressing adenovirus (Ad‐LRRC8A), or the respective negative control (Negative and Ad‐Lacz) for 48 h, the cell viability was measured by CCK‐8 assay (A); cell cycle transition was detected by flow cytometric analysis (B) (*n* = 6, *p < 0.05). (C, D) Representative western blot images (C) and the quantification graphs (D) of Cyclin D1, Cyclin E, CDK2 expression in LRRC8A siRNA‐treated BASMCs. Ctrl, control; Neg, negative control; si‐RNA, LRRC8A siRNA (*n* = 6, *p < 0.05 VS Ctrl or Neg). (E, F) Representative western blot images (E) and the quantification graphs (F) of Cyclin D1, Cyclin E, and CDK2 expressions in LRRC8A‐expressing adenovirus‐transfected BASMCs. Ctrl, control; Ad‐LacZ, LacZ control adenovirus; Ad‐LRRC8A, LRRC8A overexpression adenovirus (*n* = 5, *p < 0.05 VS Ctrl or Ad‐LacZ)

It has been demonstrated that VRAC and volume‐regulated Cl^−^ movement are strongly associated with cerebrovascular remodeling by affecting BASMCs proliferation in our previous studies.^20^ We next wanted to examine the effect of LRRC8A on BASMCs proliferation. Our results showed that siRNA‐mediated knockdown of LRRC8A significantly reduced the proliferation rate, whereas overexpression of LRRC8A increased the proliferation rate of BASMCs (Figure 4A). Flow cytometry analysis revealed that upregulation of LRRC8A promoted cell cycle transition from G0/G1 phase to S phase; the percentage of G0/G1 phase cells was decreased from 72.74 ± 1.62% to 60.11 ± 2.59%, whereas the percentage of S phase cells was increased from 15.90 ± 1.06% to 25.10 ± 0.82%. Silencing of LRRC8A exerted an opposite effect with the increased percentage of G0/G1 phase and decreased percentage of S phase (Figure 4B, Figure S3). Cell cycle transition is regulated by the multiple cell cycle regulators, including both positive regulators and negative regulators. To examine whether LRRC8A regulated the proliferation rate of BASMCs via those cell regulators, we also tested the expressions of these cell cycle regulators including Cyclin D1, Cyclin E, and CDK2 by western blot. The results revealed that Cyclin D1, Cyclin E, and CDK2 were markedly inhibited in LRRC8A siRNA treated cells, whereas overexpression of LRRC8A increased the expression of these regulators (Figure 4C–F). These data demonstrated that LRRC8A promotes proliferation via increasing the expression of positive cell cycle regulators in BASMCs.

### 3.5 LRRC8A promotes BASMCs proliferation through WNK1/PI3K‐p85/AKT signaling pathway

**FIGURE 5:**
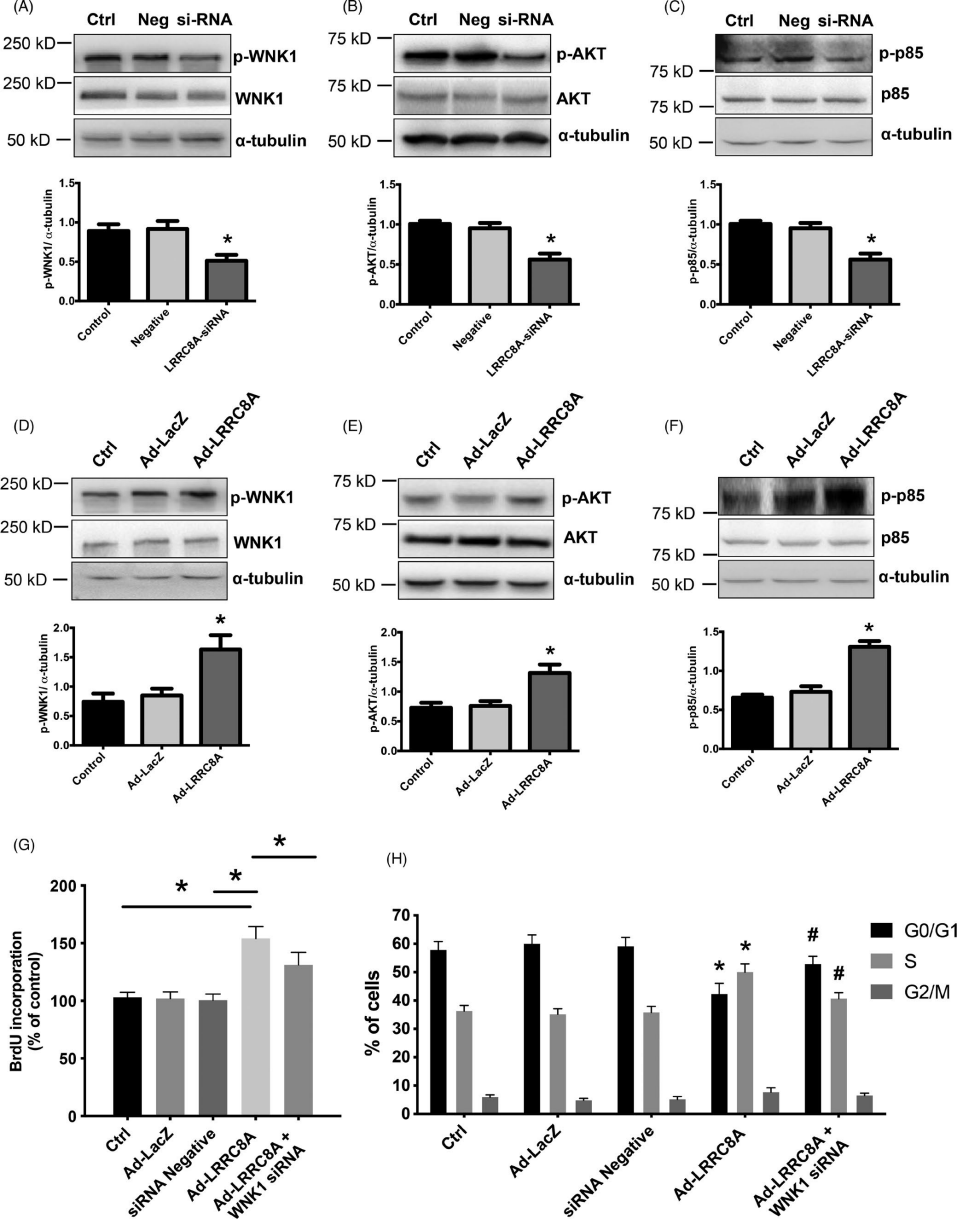
LRRC8A promotes BASMCs proliferation through WNK1/PI3K‐p85/AKT signaling axis. (A–C) Representative western blot images and the respective quantification graphs showing the expression of p‐WNK1 (A), p‐AKT (B), and p‐p85 (C) in LRRC8A siRNA‐treated BASMCs. Ctrl, control; Neg, negative control; si‐RNA, LRRC8A siRNA (*n* = 5, *p < 0.05 VS Ctrl or Neg). (D–F) Representative western blot images and the respective quantification graphs showing the expression of p‐WNK1 (D), p‐AKT (E), and p‐p85 (F) in LRRC8A siRNA‐treated BASMCs. Ctrl, control; Ad‐LacZ, LacZ control adenovirus; Ad‐LRRC8A, LRRC8A‐expressing adenovirus (*n* = 6, *p < 0.05 VS Ctrl or Ad‐LacZ). (G) BrdU incorporation assay revealed that overexpression of LRRC8A increased BASMCs proliferation, knock down of WNK1 inhibited the increased effects (*n* = 5, *p < 0.05). (H) Cell cycle transition was detected by flow cytometric analysis with indicated treatment (*n* = 4, *p < 0.05 VS Ctrl, ^#^ *p* < 0.05 VS Ad‐LRRC8A)

Our previous studies have demonstrated that WNK1 is sensitive to hypotonic stimulation‐dependent Cl^−^ transport from inside the cell to outside the cell, which leads to the activation of WNK1 through phosphorylation and PI3K‐p85/AKT signaling axis.^31^ Consequently, we investigated whether LRRC8A promoted BASMCs proliferation through activation of WNK1. The results showed that silencing of LRRC8A suppressed the phosphorylation of WNK1, AKT, and p85, whereas overexpression of LRRC8A increased the phosphorylation of WNK1, AKT, and p85 (Figure 5A–F). To further validate that WNK1 signaling cascade was responsible for LRRC8A‐mediated BASMCs proliferation, we knocked down WNK1 expression with specific siRNA and found that downregulation of WNK1 significantly reversed the increased proliferation evoked by overexpression of LRRC8A in BASMCs, as assessed by BrdU incorporation assay (Figure 5G). In addition, silencing of WNK1 reversed the cell cycle transition, as indicated by an increased proportion of cells in G0/G1 phase and a decreased proportion of cells in S phase (Figure 5H, Figure S4). The results above indicated that WNK1/PI3K‐p85/AKT was the downstream signaling axis involved in proliferation induced by upregulation of LRRC8A.

### 3.6 LRRC8A exacerbates hypertension‐induced cerebrovascular vascular remodeling

**FIGURE 6:**
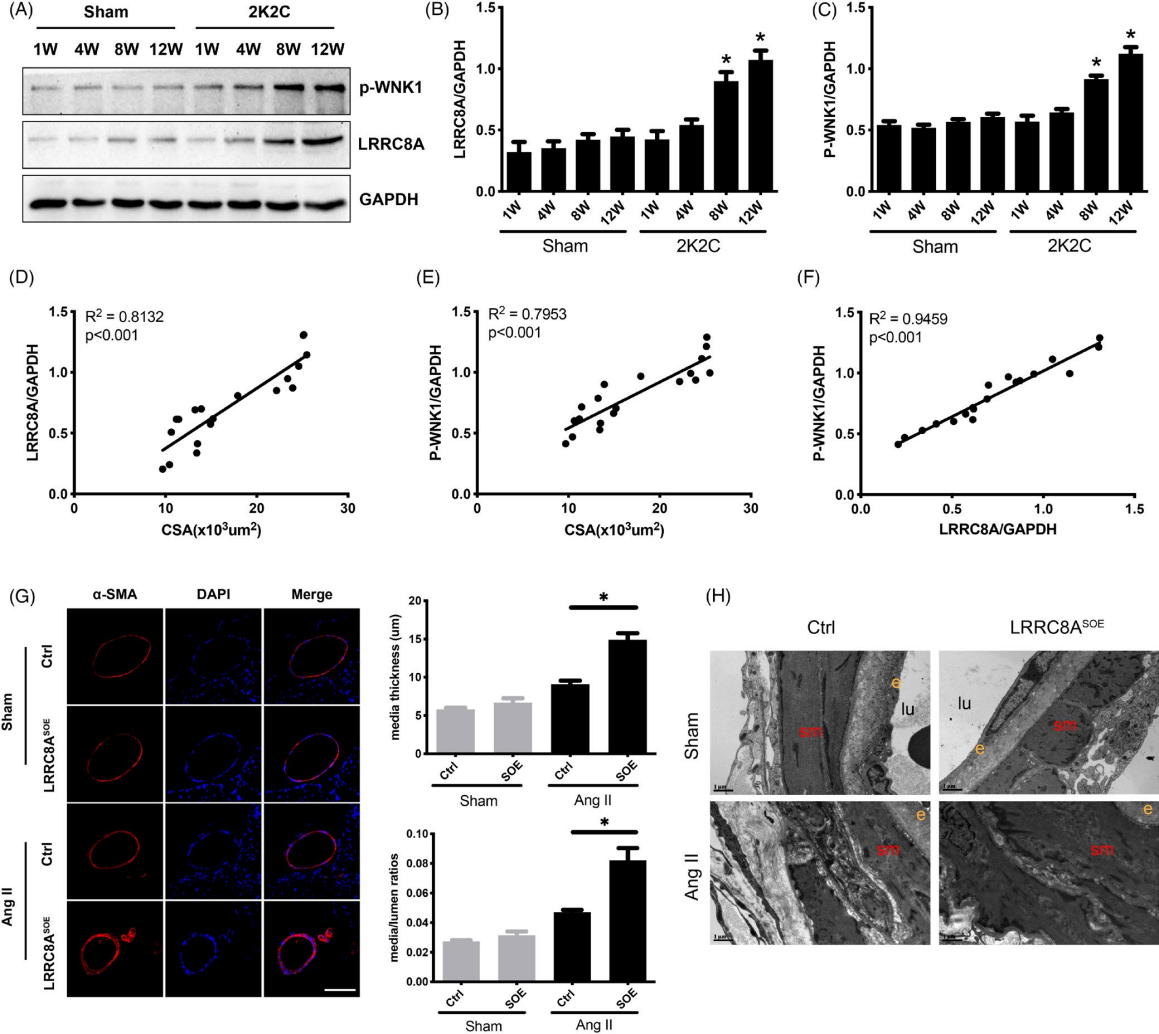
Overexpression of LRRC8A in smooth muscle cells exacerbates hypertension‐induced cerebrovascular vascular remodeling. (A–C) Representative western blot images (A) and the respective quantification graphs (B, C) showing the expression of LRRC8A and p‐WNK1 in basilar artery from 2k2c renal hypertension model at the indicated time points (*n* = 3, *p < 0.05 VS Sham). (D) Correlation between the relative expression of LRRC8A and medial cross‐sectional area (CSA) of basilar artery from 2k2c renal hypertension model. (E) Correlation between the relative expression of p‐WNK1 and medial cross‐sectional area (CSA) of basilar artery from 2k2c renal hypertension model. (F) Correlation between the relative expression of p‐WNK1 and the relative expression of LRRC8A from 2k2c renal hypertension model. (G) Representative images of α‐SMA staining and quantification graphs of basilar artery media thickness and media/lumen ratio in LRRC8A smooth muscle overexpressing (LRRC8A^SOE^) mouse with or without Ang II infusion (*n* = 5, *p < 0.05). (H) The electron microscopy images of basilar artery from LRRC8A^SOE^ mouse with or without Ang II infusion, sm, smooth muscle; e, endothelium; lu, lumen; Scale: 1 μm

Excessive proliferation of cerebrovascular cells is one of the main factors contributing to vascular remodeling during hypertension. To determine the functional relevance of LRRC8A in vascular remodeling, we firstly examined the expression level of LRRC8A in basilar artery from 2k2c renal hypertension model. Our results revealed that the expression of LRRC8A was progressively increased during the development of hypertension, accompanied by the activation of WNK1 (Figure 6A–C). Meanwhile, LRRC8A expression was positively correlated with median cross‐sectional area (CSA) and the phosphorylation of WNK1, suggesting that LRRC8A was indeed involved in the process of vascular remodeling (Figure 6D–F). To better understand the potential role of LRRC8A in vascular remodeling during hypertension, we next knocked in a construct containing a CAG promoter followed by a loxP‐Stop‐loxP‐LRRC8A coding region cassette, and we generated smooth muscle‐specific LRRC8A overexpression mice (LRRC8A^SOE^) by crossing this knock‐in mouse line with Tagln Cre mice (Figure S5). LRRC8A^SOE^ mice were implanted with Angiotensin II infusion pumps for 4 weeks starting from 8 weeks after birth. Our results indicated that Angiotensin II infusion significantly increased cerebrovascular vascular remodeling; the remodeling of basilar arteries in LRRC8A^SOE^ mice was much more severe than control mice after Angiotensin II infusion, leading to a significant morphological change, including increased media thickness and media/lumen ratio (Figure 6G). Meanwhile, the cerebrovascular vascular remodeling was further validated by electron microscopy analyses. As shown in Figure 6H, Angiotensin II infusion increased the thickness of smooth muscle layer in vessel wall, and this ultrastructure change was dramatically increased in LRRC8A^SOE^ mice. Taken together, these results suggested that smooth muscle specific overexpression of LRRC8A aggravated cerebrovascular vascular remodeling during Angiotensin II‐induced hypertension.

## 4 DISCUSSION

The molecular identity of VRAC is complicated. Although some candidates, such as P‐glycoprotein, ClC‐3, and Bestrophin‐1,^10^, ^11^, ^12^ have been reported as components of VRAC, none of them have been demonstrated to be sufficient for VRAC activity in all cell types. In 2014, the studies from two laboratories identified LRRC8A as an essential component of VRAC.^13^, ^14^ It has been demonstrated that LRRC8A homohexamers poorly recapitulated VRAC electrophysiological properties.^38^ Accumulating evidence from the structure of LRRC8A and other electrophysiological studies indicated that heteromeric channels comprising LRRC8A and at least one other LRRC8 paralog are crucial for VRAC activity.^37^, ^38^, ^39^, ^40^ However, some other studies show that LRRC8A is dispensable for VRAC activity,^15^, ^16^ suggesting that LRRC8A alone is not sufficient for volume regulation in response to hypotonic stimulation and VRAC activity in all cell types. The controversy of these studies led us to investigate whether LRRC8A is necessary for VRAC functions in cerebrovascular smooth muscle cells. Consistent with other studies, our studies found that the silencing of LRRC8A strongly inhibited VRAC current and Cl^−^ transport, whereas the overexpression of LRRC8A did not further enhance VRAC activity induced by hypotonic solution. In contrast, we unexpectedly found that the overexpression of LRRC8A evoked a voltage‐dependent Cl^−^ current in isotonic condition but did not further enhance hypotonic solution induced Cl^−^ current and Cl^−^ efflux. The treatment of Cl^−^ channel blockers significantly inhibited this current. The anion selectivity for this current was consistent with our previous studies.^23^ LRRC8A is a protein containing a transmembrane pore domain followed by a C‐terminal LRRD. Although LRRD is predicted to be involved in protein‐protein interaction in many LRRD‐containing proteins and the flexibility of the LRR domains may play a functional role in channel gating, the precise role of LRRD in LRRC8A function remains unclear. In this study, we found that overexpression of LRRC8A induced voltage‐dependent Cl^−^ current in isotonic solution, which was not observed in LRRD‐truncated LRRC8A transfected cells. One possible explanation of this interesting phenomenon is that the overexpression of LRRC8A in BASMCs could regulate cell size even under normal conditions, whereas LRRD functions as the key domain of a volume sensor. However, further studies are needed to determine the molecular mechanisms by which LRRD regulates channel gating and properties.

It has been demonstrated that VRAC is associated with cell proliferation in our previous studies. Here, we found that the overexpression of LRRC8A increased BrdU incorporation, expressions of CyclinD1, Cyclin E and CDK2, and accelerated cell cycle transition from G1 to S phase in BASMCs. Additionally, upregulation of LRRC8A increased WNK1, PI3K, and AKT phosphorylation levels. Silencing of WNK1 reduced overexpression of LRRC8A‐induced cell proliferation. These results suggest that upregulation of LRRC8A promotes BASMC proliferation through activation of WNK1, which subsequently activates the PI3K‐AKT signal pathway.

Our previous studies determine that VRAC accelerates the cerebrovascular and remodeling during the development of hypertension, and there is a positive correction between VRAC activity and cerebrovascular remodeling.^20^, ^22^ Cerebrovascular remodeling is largely contributed by BASMCs proliferation. Similar to the relation between VRAC activity and cerebrovascular remodeling during hypertension, our results demonstrated that the expression of LRRC8A was positively correlated with rat basilar artery remodeling during the development of hypertension in 2k2c hypertension models. There was a positive correlation between expression of LRRC8A and WNK1 phosphorylation levels in the development of cerebrovascular remodeling. More importantly, using the LRRC8A transgenic mice, we found that overexpression of LRRC8A in smooth muscle cells further exacerbated hypertension‐induced vascular remodeling in Angiotensin II infusion models. Whether downregulation of LRRC8A would ameliorate hypertension‐induced cerebrovascular vascular remodeling can be determined using a smooth muscle‐specific knockout model in the further studies. Altogether, results in this present study demonstrate that LRRC8A is an essential component of VRAC, LRRC8A mediates cerebrovascular cell proliferation through WNK1/PI3K‐AKT signaling pathway and plays an important role in hypertension‐induced cerebrovascular remodeling, suggesting that LRRC8A is a potential therapeutic target for vascular remodeling.

## CONFLICT OF INTEREST

The authors declare no conflicts of interest.

## AUTHOR CONTRIBUTIONS

Y.‐Y.G., C.L., and X.‐Y.L. designed the study. X.‐Y.L., X.‐F.L., and C.‐C.H. performed the experiments and analyzed data. L.S. and M.‐M.M. helped with the experiments. C.L., X.‐Y.L., and Y.‐Y.G. wrote the manuscript.

## Supporting information

Supplementary Material

## Data Availability

The data and study materials that support the findings of this study will be available to other researchers from the corresponding authors on reasonable request.
