# Supplementary material for: LRRC8A is essential for volume‐regulated anion channel in smooth muscle cells contributing to cerebrovascular remodeling during hypertension
Source: Cell Prolif. 2021 Nov 1;54(12):e13146. doi: 10.1111/cpr.13146 (PMC8666279; doi:10.1111/cpr.13146)

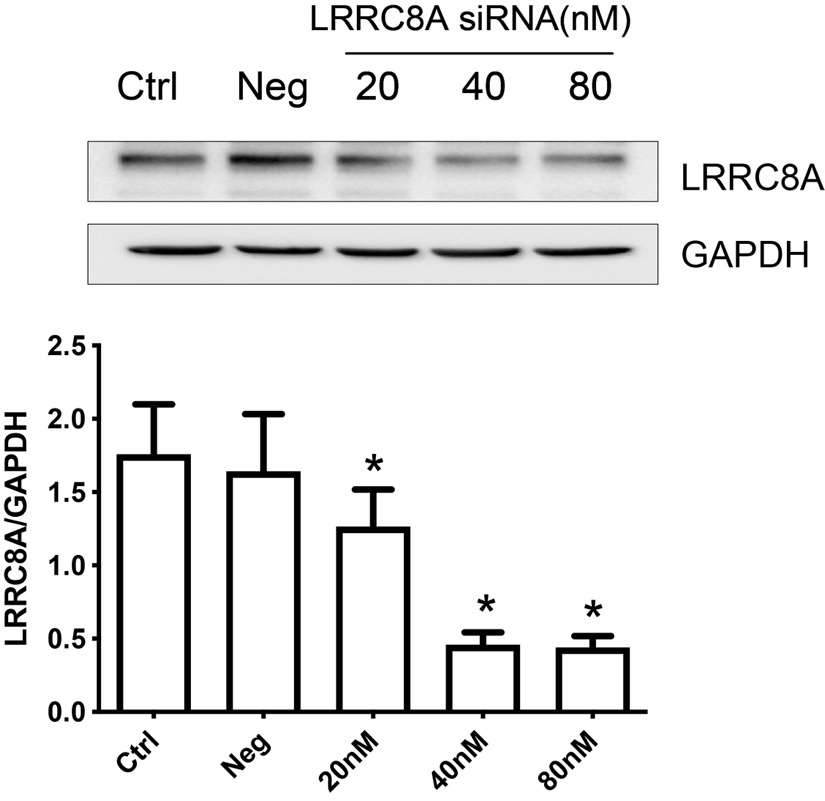


**Figure S1. Knockdown efficiency of LRRC8A siRNA in BASMCs was detected by western blot.** Representative western blot images and the respective quantification graphs showing cells were transfected with LRRC8A siRNA (40 nM) efficiently abolished the endogenous expression of LRRC8A. (n=3, *P<0.05 VS Ctrl or Neg)

**
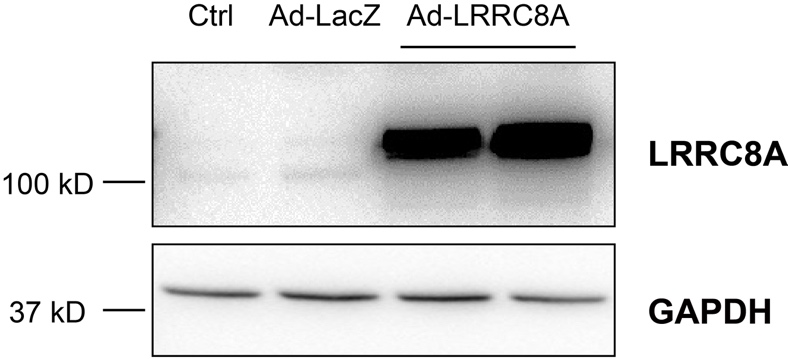
**

**Figure S2. Western blot confirmation of LRRC8A protein expression induced by adenovirus mediated overexpression of LRRC8A in BASMCs.**

**
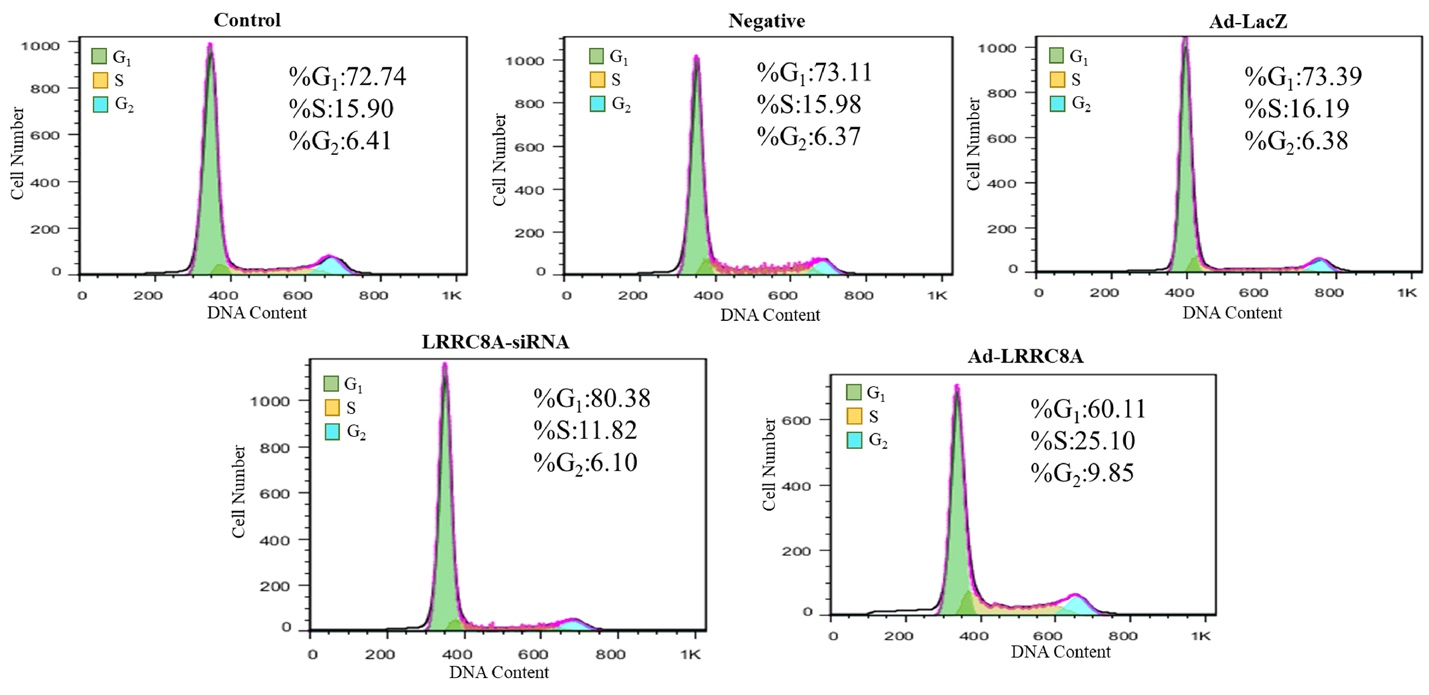
**

**Figure S3. Representative cell cycle flow cytometry diagram of Figure 4B.**

**
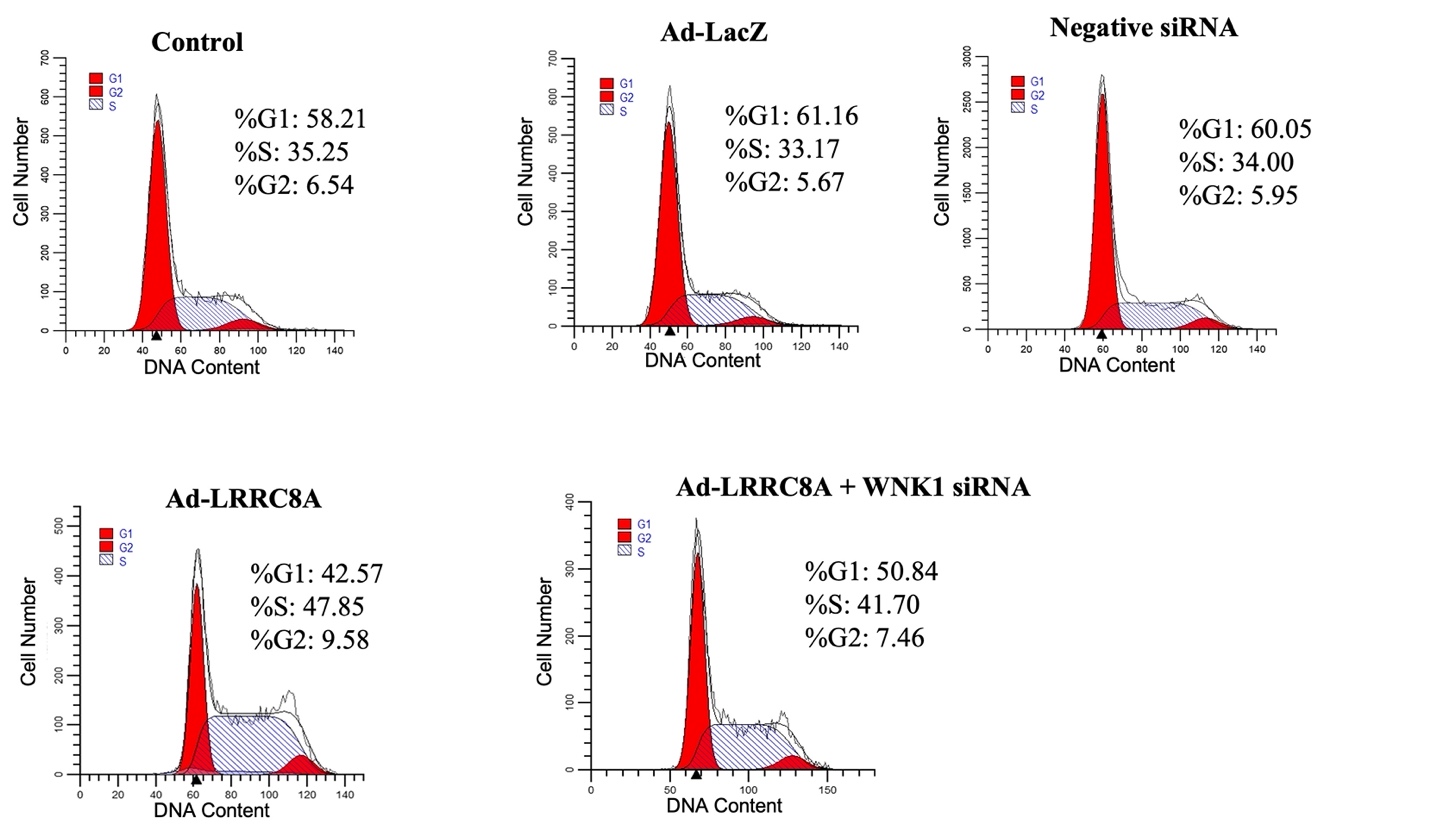
**

**Figure S4. Representative cell cycle flow cytometry diagram of Figure 5H.**


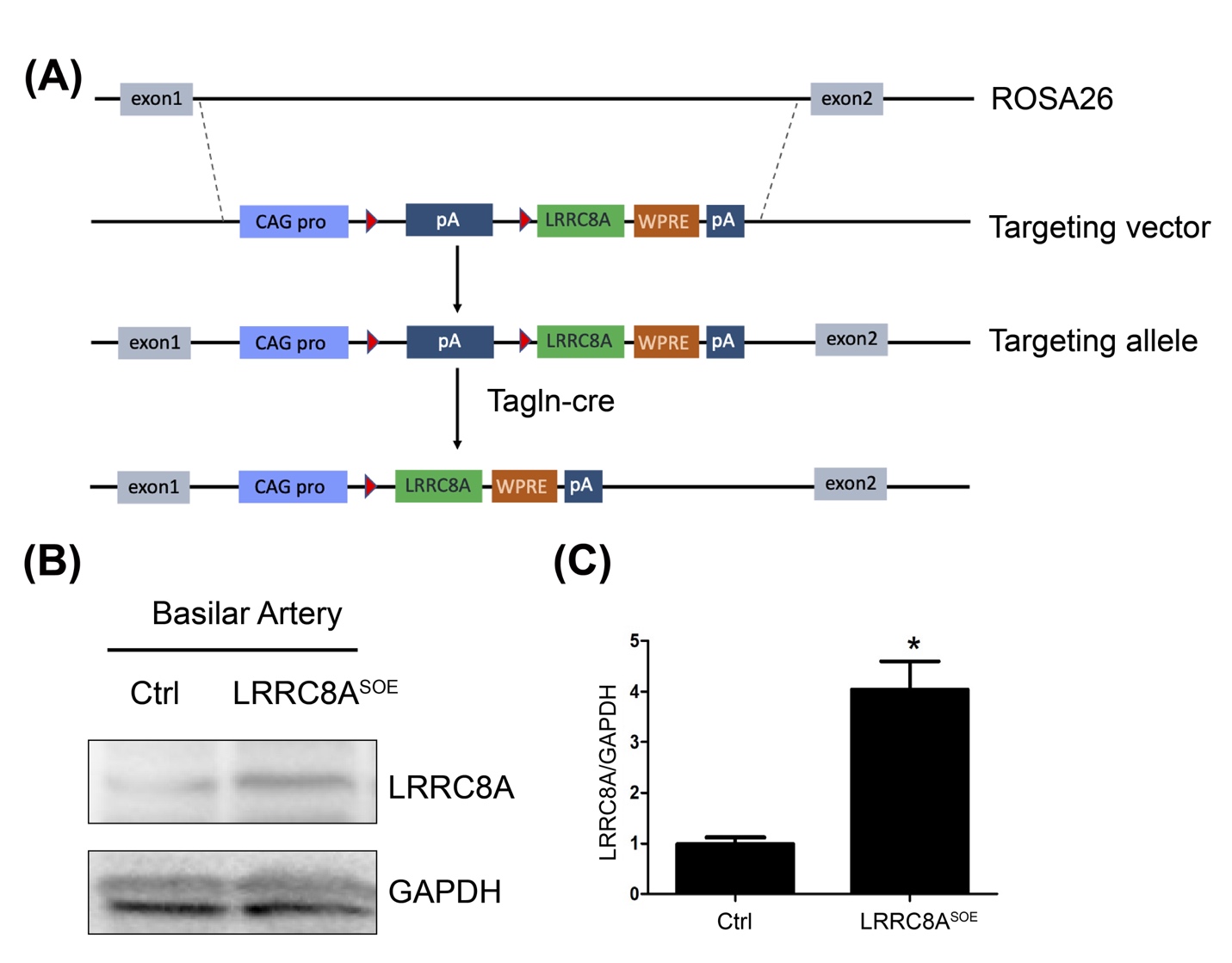


**Figure S5. Generation of LRRC8A smooth muscle specific transgenic mice.** (A) the schematic diagram of LRRC8A smooth muscle overexpression mice. (B, C) the expression of LRRC8A was validated in basilar artery by western blot. (n=4, *P<0.05 VS Ctrl)

**Supplemental Table 1. Antibodies and reagents used in this study.**

| **Antibody/reagent** | **Source, Cat. No** | **Antibody/reagent** | **Source, Cat. No** |
| --- | --- | --- | --- |
| Newborn Calf Serum | Gibco, 26010074 | Foetal Bovine Serum | Gibco, 10100147C |
| α-tublin antibody | Santa Cruz Biotechnology, sc-8035 | Phospho-PI3Kp85 Antibody | Cell Signaling Technology, 4228 |
| Cell cycle detection kit | Keygen, KGA512 | Angiotensin Ⅱ | Sigma, A9525 |
| Cyclin D1 Antibody | Cell Signaling Technology, 2922 | Cyclin E Antibody | Cell Signaling Technology, 20808 |
| CDK2 Antibody | Cell Signaling Technology, 2546 | Phospho-Akt Antibody | Cell Signaling Technology, 4060 |
| P-WNK1 antibody | SAB, 12295 | LRRC8A antibody | AVIVA SYSTEMS BIOLOGY, ARP49453_P050 |
| GAPDH | Vazyme, Ab103-01 | α-Smooth Muscle Actin antibody | BOSTER Biological Technology, BM0002 |
| Cell Proliferation ELISA, BrdU | Roche, 11647229001 | LRRC8A antibody (FITC) | biorbyt, orb189367 |

**Supplemental Table 2. The primers used for genotyping.**

| Target gene Primers | 5’-3’ | Application |
| --- | --- | --- |
| LRRC8A-F | CAACGTGCTGGTTATTGTGCTGTCT | Mice genotyping |
| LRRC8A-R | GCATGACGATGGAGATGTAATCAGTG |  |

**Supplemental Table 3. The reversal potential (mV) induced by different anions and** **their relative permeability (permeability ratio of anions).**


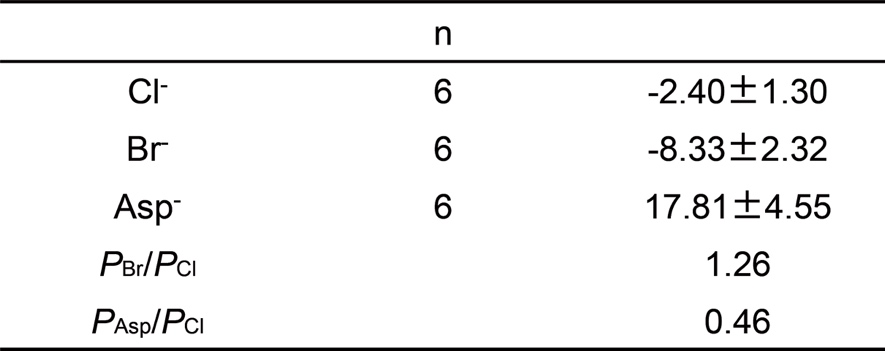

Supplement: Supplementary file 1 — Supplementary Material [file CPR-54-e13146-s001.docx]
